# Protein Nanoparticles Modified with PDGF-B as a Novel Therapy After Acute Cerebral Infarction

**DOI:** 10.1523/ENEURO.0098-21.2021

**Published:** 2021-09-15

**Authors:** Soh Takagishi, Koichi Arimura, Masaharu Murata, Katsuma Iwaki, Tomohiro Okuda, Keisuke Ido, Ataru Nishimura, Sayoko Narahara, Takahito Kawano, Koji Iihara

**Affiliations:** 1 Department of Neurosurgery, Graduate School of Medical Sciences, Kyushu University, Fukuoka 812-8582, Japan; 2 Center for Advanced Medical Innovation, Kyushu University, Fukuoka 812-8582, Japan; 3 Department of Advanced Medical Initiatives, Faculty of Medical Sciences, Kyushu University, Fukuoka 812-8582, Japan; 4 National Cerebral and Cardiovascular Center, Suita, Japan, Osaka 564-8565, Japan

**Keywords:** cerebral infarction, nanoparticle, neuroprotection, pericyte, platelet-derived growth factor PDGF-B

## Abstract

Treatment options for cerebral infarction beyond the time window of reperfusion therapy are limited, and novel approaches are needed. PDGF-B is considered neuroprotective; however, it is difficult to administer at effective concentrations to infarct areas. Nanoparticles (NPs) are small and stable; therefore, we modified PDGF-B to the surface of naturally occurring heat shock protein NPs (HSPNPs) to examine its therapeutic effect in cerebral infarction. PDGF-B modified HSPNPs (PDGF-B HSPNPs) were injected 1 d after transient middle cerebral artery occlusion (t-MCAO) in CB-17 model mice. We analyzed the infarct volume and motor functional recovery at 3 and 7 d. PDGF-B HSPNPs were specifically distributed in the infarct area, and compared with HSPNPs alone, they significantly reduced infarct volumes and improved neurologic function 3 and 7 d after administration. PDGF-B HSPNP administration was associated with strong phosphorylation of Akt in infarct areas and significantly increased neurotrophin (NT)-3 production as well as reduced cell apoptosis compared with HSPNPs alone. Moreover, astrogliosis in peri-infarct area was significantly upregulated with PDGF-B HSPNPs compared with HSPNPs alone. Treatment with PDGF-B HSPNPs might be a novel approach for treating cerebral infarction.

## Visual Abstract

**Figure:**
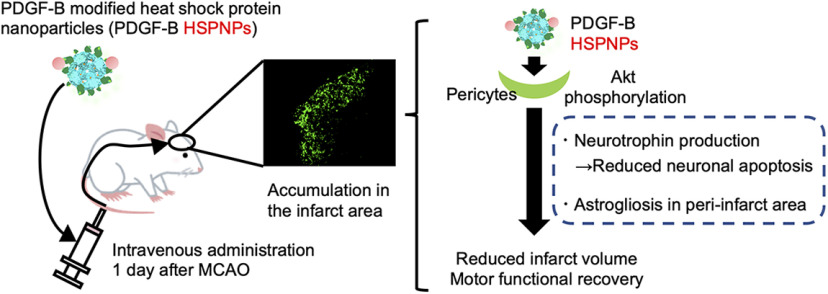

## Significance Statement

This study involved injection of PDGF-B protein-modified heat shock protein nanoparticles (PDGF-B HSPNPs) 1 d after transient middle cerebral artery occlusion (t-MCAO) in CB-17 model mice. We demonstrated that PDGF-B HSPNPs significantly reduced the infarct volume and improved motor functional recovery at 3 and 7 d after administration. We also demonstrated that phosphorylation of Akt and increased in pericytes and expression of neurotrophin (NT)-3 was promoted with PDGF-B HSPNPs, which may lead to reduce apoptotic neuronal death in peri-infarct area. Additionally, it has been revealed that astrogliosis in peri-infarct area was also promoted with PDGF-B HSPNPs. These results support the use of PDGF-B HSPNPs as a novel therapeutic approach for ischemic stroke.

## Introduction

Ischemic stroke is a major cause of disability. The efficacy of early reperfusion therapy for acute ischemic stroke with tissue plasminogen activator (t-PA; The National Institute of Neurological Disorders and Stroke rt-PA Stroke Study Group, 1995; Hacke et al., 2008) and mechanical thrombectomy with a stent retriever (Goyal et al., 2016) has been established by randomized controlled trials. Moreover, it has been shown that mechanical thrombectomy was effective up to 16–24 h after stroke onset in selected cases, based on perfusion imaging (Nogueira et al., 2018; Albers et al., 2018). Although these treatments are effective in some patients, the number of eligible candidates for reperfusion therapy is low because of its narrow therapeutic time window. The standard treatment for ischemic stroke beyond the time window of reperfusion therapy is limited to the prevention of recurrence with antithrombotic agents and rehabilitation, but disability is usually permanent in these patients. Therefore, novel therapeutic approaches for patients with ischemic stroke, beyond reperfusion therapy, are urgently required.

The pericyte is a type of cell found in neurovascular units, which encircles endothelial cells in capillary vessels and plays important roles in angiogenesis, vessel maturation, and stabilization (Armulik et al., 2011) as well as maintenance of the blood-brain barrier (BBB; Armulik et al., 2010), regulation of blood flow (Hall et al., 2014), regulation of antioxidants (Nishimura et al., 2016), repair of damaged tissue (Makihara et al., 2015), and regulation of neurotrophic factors (Arimura et al., 2012). Additionally, previous studies have shown that pericytes are activated through PDGF-B signaling 3–7 d after ischemic stroke in rodent models (Arimura et al., 2012; Makihara et al., 2015). Therefore, we focused on PDGF-B signaling in pericytes as a therapeutic target for cases beyond the time window of reperfusion therapy.

It has been reported that pretreatment with PDGF-B using osmotic pumps plays a protective role in ischemic stroke by preventing delayed neuronal death and inducing infarct tolerance (Iihara et al., 1997; Sakata et al., 1998). However, the same has not been reported regarding treatment with PDGF-B after ischemic stroke. This may be because of difficulty in achieving selective accumulation and prolonged action of PDGF-B in the area of infarction. For this reason, we focused on a drug delivery system (DDS) based on nanoparticles (NPs). It is well known that NPs accumulate in regions with damaged vasculature because of the enhanced permeability and retention effect. Given previous reports that NPs accumulate in areas of myocardial infarction (Yao et al., 2015), we hypothesized that NPs may be able to carry PDGF-B to the infarct area in ischemic stroke.

Generally, nano-sized particles are very small and stable; therefore, they accumulate in cerebral infarcts and may remain there for a long time without being metabolized. As a model NP, we focused on small heat shock protein 16.5 (HSP), a naturally occurring protein in *Methanococcus jannaschii*, which forms an NP structure with an outer diameter of 12 nm by self-assembly of 24 subunits (Kim et al., 1998, 2003; Koteiche et al., 2005). As a stable NP that circulates in the blood, HSP-based nanomaterials possess good biocompatibility and adequate stability. The design, synthesis, and biological characterization of several types of protein NPs have been reported (Toita et al., 2012, 2013; Murata et al., 2015; Hamano et al., 2016; Kawano et al., 2018). HSPNPs are attractive as a biomedical tool for drug delivery or imaging because of their biocompatibility, monodispersed formation, robust structure, easy acquisition from *Escherichia coli*, and simple functionalization through chemical and genetic strategies. Therefore, targeting HSPNPs to certain cells or tissues is very important for disease treatment and diagnostic imaging. In this study, we investigated the effect of PDGF-B in ischemic stroke using PDGF-B modified protein NPs as a drug carrier targeting the infarct area and its detailed therapeutic mechanisms.

## Materials and Methods

### Expression and purification of HSPNPs

The pET21a(+) vector-encoded HSPG41C mutant, in which the Gly 41 residue of wild-type HSP was substituted with a Cys residue, was prepared by polymerase chain reaction-mediated mutagenesis using appropriate primers (Toita et al., 2012, 2013). Successful mutagenesis was confirmed by DNA sequencing. The HSPG41C mutant protein was obtained from *E. coli* and was purified by anion-exchange chromatography and size-exclusion chromatography (SEC). The BL21 (DE3) strain of *E. coli* (Merck KGaA) was used for HSPG41C expression. *E. coli* containing the pET21a (+) plasmid vector was grown in 2 × YT medium containing 100 μg/ml of ampicillin at 37°C. When the culture medium’s optical density at 600 nm reached 0.5−0.6, the expression of the recombinant protein was induced by exposure to 1 mm isopropyl-β-D-thiogalactopyranoside (Wako Pure Chemical Industries) for 4 h at 37°C. After cell harvesting by centrifugation, cells were suspended in 25 mm monopotassium phosphate solution containing 1 mm EDTA and 2 mm dithiothreitol, pH 7.0, and stored at −80°C pending purification. The cell suspensions were then sonicated to disrupt the cell membrane. The resulting cell lysates were centrifuged at 15,000 × *g* for 30 min at 4°C, and the supernatant was collected. Proteins were purified using a HiPrep Q HP 16/10 anion exchange column (GE Healthcare) and a SEC column (TSKgel G4000SW; Tosoh Corporation). Successful purification was confirmed by electrophoresis with 15% SDS-PAGE.

### Synthesis of PDGF-B modified HSPNPs (PDGF-B HSPNPs)

To introduce a chelating function to the surface of HSPG41C, isothiocyanobenzyl-NTA (*N*-[5-(4-isothiocyanatobenzyl)amido-1-carboxypentyl]iminodiacetic acid; Dojindo Laboratories) was used. Through the NTA moiety attached to the surface, a genetically edited protein bearing a hexahistidine extension (His-tag) at its terminus can be immobilized to the surface via chelation bridging through nickel (II). HSPNPs were modified through amide bond formation with isothiocyanobenzyl-NTA using a 20 × excess of the protein monomer in HEPES (0.1 m, pH 8.5) for 24 h at room temperature, followed by removal of unreacted isothiocyanobenzyl-NTA using ultrafiltration. Successful modification of the HSPNPs was confirmed with matrix assisted laser desorption ionization-time of flight MS. For modification of PDGF-B, recombinant human PDGF-B protein bearing a His-tag (SPEED BioSystems) was prepared. HSPNP modified-NTA and PDGF-B protein (2 equiv. to HSPG41C) were dissolved in HEPES (0.1 m, pH 8.0) with 1 mm nickel (II) and stirred for 24 h at 4°C. Unreacted protein was removed by ultrafiltration with a molecular weight cutoff of 100,000.

### NP characterization

The hydrodynamic diameter of the NPs (10 μmol/l) was measured using dynamic light scattering at a detection angle of 173° and a temperature of 25°C using a Zetasizer Nano ZS Analyzer (Malvern Instruments). The 633 nm line of a helium-neon laser was used as the incident beam. All samples and buffer solutions were filtered using Ultrafree-MC centrifugal filter units with 0.22-μm pores (Millipore) before analysis. NPs were applied to carbon-coated copper grids to prepare samples for analysis using TEM. After adsorption of the NPs, the grids were rinsed with droplets of deionized water and stained with 2% uranyl acetate.

### *In vitro* experiments

Human brain pericytes isolated from normal adult human cortices were purchased from ScienCell Research Laboratories. The cells were plated on poly-D-lysine plates and cultured in pericyte medium supplemented with 10% fetal bovine serum and pericyte growth supplement at 37°C in 5% CO_2_ in a humidified incubator. Cells at passages 4–8 were used for experiments. To elucidate the role of PDGF-BB, we incubated cultured human brain pericytes with PBS (*n* = 5, 20 μl), HSPNPs (*n* = 5, 10 ng/ml, 20 μl), PDGF-BB (*n* = 5, 10 ng/ml, 20 μl), or PDGF-B HSPNPs (*n* = 5, 10 ng/ml, 20 μl) at 37°C for 10 min. We extracted the proteins from the cultured cells for immunoblot analysis.

### Immunoblot analyses

Cultured cells and brain tissue were homogenized in RIPA lysis buffer (Wako), phosphatase inhibitor cocktail (Thermo Fisher Scientific), and protease inhibitor cocktail (Cell Signaling Technology Japan). Protein concentration was determined using a microplate BCA protein assay kit (Thermo Fisher Scientific). The samples were subjected to Super Sep Ace (20 μg/lane, Fujifilm) and then transferred onto PVDF membranes. The membranes were incubated at room temperature with ECL prime blocking agent (GE Healthcare) for 1 h and probed with primary antibodies to Akt, phospho-Akt (Ser 473; all from Cell Signaling Technology Japan), and β-actin (Sigma) at 4°C overnight. The membranes were then washed and incubated for 1 h at room temperature with the secondary antibody (1:2000 dilution, Cell Signaling Technology Japan). Blots were developed using the ECL prime Western blot detection reagent (GE Healthcare) according to the manufacturer’s instructions.

### Measurement of neurotrophic factors

The concentrations of neurotrophic factors in protein samples from infarcted brain tissue were measured with a multi-neurotrophin (NT) rapid screening ELISA kit for NGF, BDNF, NT-3, and NT-4/5 (Biosensis Pty Ltd.), according to the manufacturer’s instructions. Absorbance was measured at 450 nm with a microplate reader (Bio-Rad).

### Mouse stroke model

All animal procedures were performed in accordance with the Kyushu University animal care committee’s regulations. All animal experiments complied with the Animal Research: Reporting In Vivo Experiments (ARRIVE) guidelines. CB-17 (CB-17/lcr-+/+Jcl) wild-type mice were purchased from CLEA Japan. We used both male and female mice aged 7–19 weeks and weighing 18–30 g. A transient middle cerebral artery occlusion (t-MCAO) model mouse was created using the following method (Kasahara et al., 2013). We used this mouse stroke model because individual differences of ischemic volume might be small because of less collateral circulation in CB-17 strain compared with other mouse strains, and we could focus on the effect of NPs. The mice were randomly assigned to surgery and administered intraperitoneal anesthesia with ketamine (100 mg/kg) and xylazine (10 mg/kg). We maintained rectal temperatures at 37°C with a heating pad. Following a midline skin incision between the left orbit and the external auditory canal, we dissected the temporal muscle to expose the temporal bone. A burr hole was made using a microdrill to expose the MCA. A 6–0 nylon suture was then threaded under the MCA, distal to the olfactory tract. The suture was rotated 180° clockwise, horizontally with the artery, for complete interruption of blood flow. The mice were returned to cages controlled at 35–37°C without anesthesia until reperfusion. After occlusion for 2 h, anesthesia was again induced and cerebral blood flow was restored by rotating the suture counterclockwise, back to its original position. The number of mice used in each experiment and related information is shown in Table 1. The following conditions excluded mice from the analyses: (1) premature death (*n* = 6); (2) hemorrhagic infarction on MRI (*n* = 2); or (3) failure of surgery and incomplete reperfusion (*n* = 2).

**Table 1:** Numbers of biological replicates

Experiment	Drug administered	Experiment day	Numbers of animals analyzed/used (sex)	Body weight (g)
MRI	PBS	7	5/6* (male)	26.4 ± 2.0
	HPSPNP	3	11/12** (male) 2/2 (female)	26.7 ± 0.7 18.7 ± 0.3
		7	6/7** (male) 2/2 (female)	26.7 ± 0.9 18.6 ± 0.1
	PDGF-BB	7	5/5 (male)	27.0 ± 1.8
	PDGF-B HSPNP (low)	7	5/5 (male)	24.1 ± 1.0
	PDGF-B HSPNP	3	13/13 (male) 2/2 (female)	27.0 ± 2.5 18.2 ± 0.2
		7	8/9* (male) 2/2 (female)	27.6 ± 2.4 18.1 ± 0.2
Cylinder test	PBS	7	5/6* (male)	26.4 ± 2.0
	HPSPNP	3	8/9** (male)	26.5 ± 0.4
		7	5/6** (male)	26.3 ± 0.4
	PDGF-BB	7	5/5 (male)	27.0 ± 1.8
	PDGF-B HSPNP (low)	7	5/5 (male)	24.1 ± 1.0
	PDGF-B HSPNP	3	12/12 (male)	26.9 ± 2.6
		7	8/9** (male)	27.6 ± 2.4
Western blotting	HSPNP	3	2/2 (male)	27.0 ± 0.3
		7	3/3 (male)	27.2 ± 1.0
	PDGF-B HSPNP	3	2/2 (male)	23.5 ± 1.1
		7	4/4 (male)	25.7 ± 1.9
Immunostaining	HSPNP	3	2/2 (male)	26.3 ± 0.0
sample		7	5/5 (male)	26.3 ± 0.4
	PDGF-B HSPNP	3	4/4 (male)	25.9 ± 2.5
		7	5/5 (male)	25.3 ± 2.1
Pilot behavioral experiment using the IVIS		1, 3, 7, 15	25/32 (male) (*, 4; **, 1; ***, 2)	25.1 ± 2.8
	Cumulative total		161/176	

Body weight values are shown as mean ± SD. The exclusion criteria were as follows: *, premature death; **, hemorrhagic infarction on MRI; ***, failure of surgery. PBS, vehicle; HSPNPs, heat shock protein nanoparticles; PDGF-BB, dimer of PDGF B; PDGF-B HSPNPs, PDGF B conjugated to HSPNPs, dose = 1.04 μmol/l; PDGF-B HSPNPs (low), same but dose = 0.104 μmol/l.

### *In vivo* MRI

MRI was performed using a 9.4 T Biospec MRI scanner (Bruker). Before the experiment, mice were anesthetized with 1.6% isoflurane in 70% N_2_O and 30% O_2_, fixed in an MR holder and placed in a 3.8 cm-diameter birdcage coil. t-MCAO model mice were imaged with MRI 1, 4, and 8 d after surgery. We captured T2-weighted images of mice and measured the ischemic volume (Wild et al., 2000) using an image analysis system (NIH ImageJ; repetition time = 3000 ms, echo time = 33 ms, number of signals averaged = 8, matrix resolution = 128 × 80, slice thickness = 1 mm, interslice distance = 1 mm, number of slices = 15–20). The infarct volume was calculated with the following formula: infarct volume ratio = (ipsilateral T2 high intensity area)/(contralateral hemisphere × 2). Because of individual differences in the cerebral infarct volume, we evaluated the infarct volume before injection of NPs as a control.

### Cylinder test

Because the degree of paresis was small, we used the cylinder test for behavioral evaluation (Li et al., 2004). Cylinder tests were performed on the day of surgery and on days 1, 4, and 8 after surgery. The mouse was placed in an acrylic cylinder, 9 cm in diameter and 20 cm in height, and recorded with a video camera. Two mirrors were placed behind the cylinder and movements of the forelimbs were recorded. The contacts of the forelimbs with the wall were recorded, and when both forelimbs were used, it was considered as “both.” However, if the right forelimb slipped after the simultaneous use of both forelimbs, it was recorded as “both” and “left.” A total of 20 sets were recorded to complete the test. Score = (left forelimb exercise – right forelimb exercise)/(left forelimb exercise + right forelimb exercise + both exercises). Under normal conditions, the score was 0 on average.

### Accumulation of HSPNPs in infarct area

PDGF-B HSPNPs were fluorescently labeled with Alexa Fluor 488-maleimide (Alexa Fluor- maleimide; 1 equiv to protein monomer; Invitrogen) in 0.1 m phosphate buffer (pH 7.2) for 24 h at 4°C. Unreacted Alexa Fluor was removed by ultrafiltration using Amicon Ultra Centrifugal Filters with a molecular weight cutoff of 100,000 (Merck KGaA) and a Zeba spin column (Thermo Fisher Scientific). Fluorescently labeled HSPNPs were prepared using the Michael addition reaction, which involves the formation of a stable bond between maleimide and a thiol group under physiological conditions. To investigate the effects of the dose of PDGF-B HSPNPs, concentrations of 0.104 and 1.04 μmol/l were used, based on previous reports. One day after surgery, PDGF-B HSPNPs (concentration of 0.104 μmol/l: *n* = 5, 0.2 ml; concentration of 1.04 μmol/l: *n* = 13, 0.2 ml), PBS (*n* = 5, 0.2 ml), HSPNPs (concentration of 1.04 μmol/l: *n* = 12, 0.2 ml), and PDGF-BB protein (concentration of 1.04 μmol/l: *n* = 5, 0.2 ml) were intravenously injected into the tail vein of t-MCAO mice. We administered PDGF-BB protein at the maximum amount, assuming that PDGF-B was attached to all HSPNPs. We determined the dose and administration time based on preliminary experiments. The mice were exsanguinated on days 3, 7, and 15 after drug administration and transcardially perfused with 20 ml of ice-cold, non-heparinized saline and 20 ml of 4% paraformaldehyde. Mouse brains were sliced into 2-mm-thick coronal sections using a mouse brain slicer (Muromachi). Thereafter, fluorescence was measured using an in vivo imaging system (IVIS; PerkinElmer). Some slices were stained with 2,3,5-triphenyltetrazolium chloride (TTC; Wako). In addition, the infarct area, which was unstained after TTC staining, was isolated, homogenized, and used for immunoblotting analyses.

Brains of t-MCAO model mice on days 3 and 7 after the injection of PDGF-B HSPNPs were embedded in optimal cutting temperature compound (Sakura Finetek), and then frozen in dry ice/ethanol. Frozen sections (10 μm) were prepared using a cryostat microtome (HM 505E, Microm). The slides were observed with a BIOREVO BZ-9000 fluorescence microscope (Keyence Corporation). Green fluorescence emitted from PDGF-B HSPNPs was detected at a 488-nm excitation wavelength and a 507-nm emission wavelength.

Immunostaining was performed using the VECTASTAIN ABC kit (Funakoshi); 2-mm-thick coronal sections were fixed with 4% paraformaldehyde in PBS for immunohistochemistry. Paraformaldehyde-fixed coronal sections were embedded in paraffin and cut into 4-μm-thick slices. The sections were then deparaffinized and activated with microwaves in 10 mmol/l Na citrate or 1 mmol/l EDTA for 10–15 min. The sections were immersed in 0.3% hydrogen peroxide-methanol solution and peroxidase-blocked for 30 min. The sections were then blocked for 1 h at room temperature with TBS-T containing 5% goat serum albumin. The sections were incubated with the following primary antibodies: anti-phospho-Akt (CST), anti-microtubule-associated protein 2 (MAP-2; Abcam) and overnight at 4°C. The mixture was incubated with biotinylated affinity-purified anti-rabbit IgG as a secondary antibody for 30 min at room temperature. The enzyme reagent reaction was conducted at room temperature for 30 min with ABC reagent. The slides were incubated with DAB until an appropriate staining intensity was obtained. The slides were observed with a BIOREVO BZ-9000 fluorescence microscope (Keyence Corporation). The sum of positive cells was measured in five randomly selected sections (60×) in the peri-infarct area (inside the MCA area of the bregma slice) and the ischemic core. The infarct volume ratio was evaluated based on the following formula: infarct volume ratio = (ipsilateral MAP-2-negative area or T2 high intensity area)/(contralateral hemisphere × 2). MAP-2-negative areas and infarct volumes were quantified using ImageJ in a blinded manner.

To examine the distribution of phosphorylated Akt in the cerebral infarction, immunofluorescent double-labeling was performed. Staining was performed using VECTASTAIN ABC kit (Funakoshi). Sections were incubated with the primary antibody anti-phospho-Akt (1:100, CST) followed by incubation with the fluorescence-labeled secondary antibody anti-PDGFRβ (1:50, CST). Then, the slides were washed and mounted with Vectashield mounting medium with DAPI (Vector Laboratories) for nuclear staining. The slides were imaged with a BZ-9000 fluorescence microscope (Keyence Corporation).

To examine the fibrotic response in peri-infarct area, immunofluorescent staining of GFAP was performed. Staining was performed using the VECTASTAIN ABC kit (Funakoshi). Sections were incubated with primary antibody GFAP (1:50, CST) and then with fluorescently labeled secondary antibody Alexa Fluor 594 goat anti-rabbit IgG (1:250, Thermo Fisher Scientific). The slides were imaged with a BZ-9000 fluorescence microscope (Keyence Corporation). Peri-infarct GFAP-positive areas were calculated using three sections. The sum of positive cells was measured in five randomly selected sections (60×) in the peri-infarct area (inside the MCA area of the bregma slice). Results are presented as cells/mm^2^.

### Terminal deoxynucleotidyl transferase-mediated biotinylated UTP nick end labeling (TUNEL) staining

To assess apoptosis in cerebral infarctions, a TUNEL assay was conducted with a direct MEBSTAIN Apoptosis TUNEL kit (MBL) based on the manufacturer’s instructions. Then, the slides were washed and mounted with Vectashield mounting medium with DAPI (Vector laboratories) for nuclear staining. The percentage of TUNEL-positive cells in the cerebral infarct was measured by counting cells using the BIOREVO BZ-9000 microscope. In addition, fluorescent/TUNEL double staining was performed to examine cells leading to apoptosis. For the primary antibody, we used the same mouse monoclonal antibody against NeuN (1:50) that we had used for immunohistochemistry at different concentrations. The secondary antibody was Alexa Fluor 594 goat anti-rabbit IgG (1:250, Thermo Fisher Scientific). The images were acquired using a BIOREVO BZ-9000 fluorescence microscope (Keyence Corporation). Green fluorescence emitted from TUNEL-positive cells was detected at a 488 nm excitation wavelength and a 507 nm emission wavelength. The sum of TUNEL-positive, TUNEL/NeuN double-positive, and NeuN-positive cells was counted in five randomly selected sections (60×) in the peri-infarct area and the ischemic core. Results are presented as cells/mm^2^.

### Statistical analysis

Data are expressed as mean ± standard deviation. Statistical analyses were performed using the unpaired *t* test with the JMP software (version 14; SAS Institute); *p* < 0.05 was considered statistically significant.

## Results

### Characterization of the engineered protein NPs

**Figure 1.**
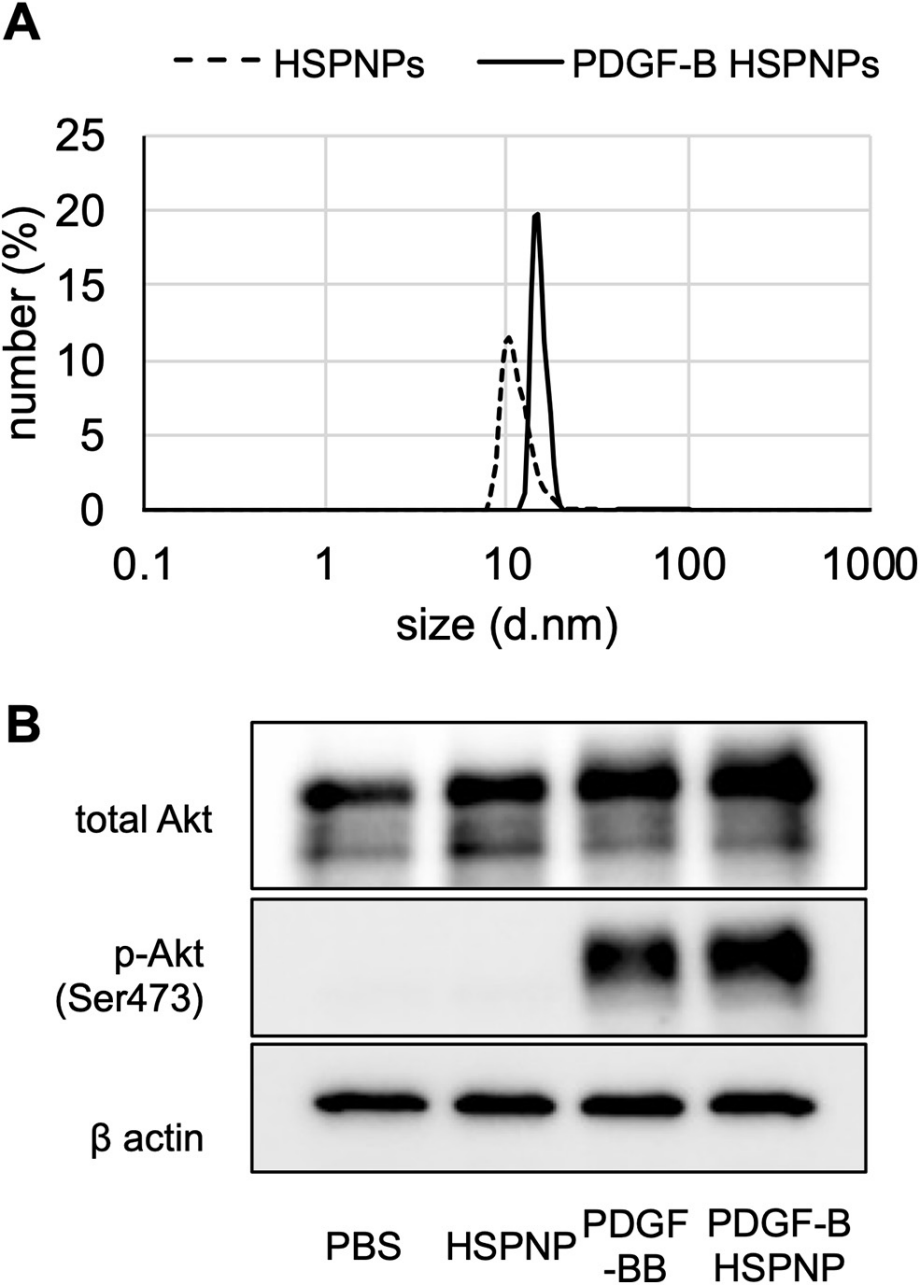
Characterization of PDGF-B HSPNP. ***A***, The size of small HSP 16.5 NPs (HSPNPs) and PDGF-B modified HSPNPs (PDGF-B HSPNPs), determined using dynamic light scattering. ***B***, Immunoblot analyses for Akt, phospho-Akt, and β-actin in cultured human brain vascular pericyte cells treated with PBS, HSPNPs, PDGF-BB, and PDGF-B HSPNPs. β-Actin was used as the loading control. d: diameter.

HSPNPs are known to form NPs with diameter of 12 nm (Kim et al., 1998; Murata et al., 2015; Kawano et al., 2018). As shown in Figure 1*A*, the HSPNPs used in the present study showed a comparable size. After conjugation of the PDGF-B protein to the HSPNPs, the size distribution peak slightly increased, but both HSPNPs were highly monodisperse and stable in an aqueous medium and did not form large aggregates.

We examined the effect of PDGF-B HSPNPs on cultured human brain vascular pericytes, following the addition of wild-type HSPNPs, PDGF-BB protein, or PDGF-B HSPNPs. Although the expression of total Akt was similar in each administration group, no phosphorylation was observed in the wild-type HSPNP group, and significant phosphorylation was observed in the PDGF-BB protein and PDGF-B HSPNP groups (Fig. 1*B*).

### PDGF-B HSPNP accumulation in the infarct area

**Figure 2.**
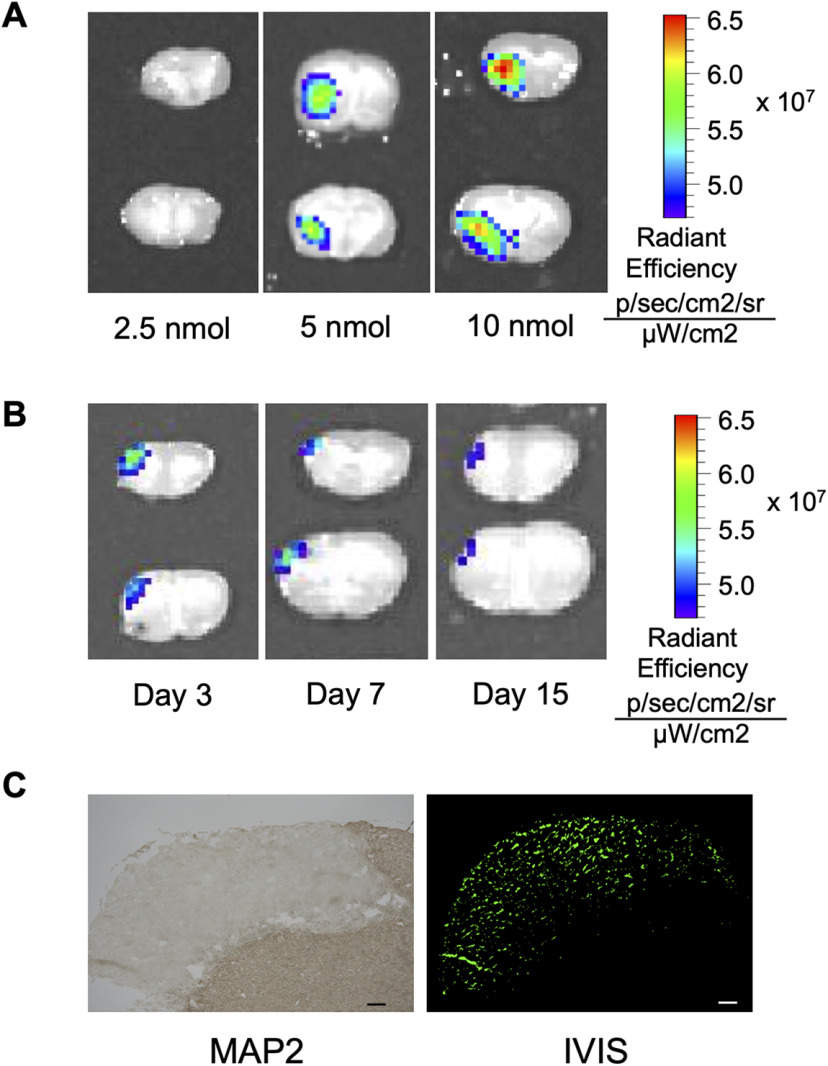
Dose and time effects of PDGF-B HSPNP accumulation in the infarct area. ***A***, Dose effects of PDGF-B HSPNP accumulation in the infarct area 6 h after administration (2.5, 5, and 10 nmol; *n* = 3–10). ***B***, Temporal profile of PDGF-B HSPNP accumulation (3, 7, 15 d after administration of PDGF-B HSPNPs; *n* = 3–10). ***C***, Frozen sections 3 d after PDGF-B HSPNP administration. Left panel, MAP-2 staining to confirm the infarct area. Right panel, Fluorescence microscopy. Scale bars: 200 μm. NPs are widely distributed in the infarct area on day 3.

To examine the accumulation and duration of PDGF-B HSPNPs in the infarct area, we injected fluorescently labeled PDGF-B HSPNPs (200 μl) into MCAO mice via the tail vein at 24 h following transient MCAO. We determined the dose and administration time based on preliminary experiments (Fig. 2*A*,*B*). Regarding the dose, accumulation in the cerebral infarct was confirmed when 5 nmol was administered (Fig. 2*A*). Accumulation of PDGF-B HSPNPs (5 nmol) was clearly confirmed in the infarct areas on days 3 and 7 and decreased slightly on day 15 (Fig. 2*B*). Brain sections were also frozen and photographed with a fluorescence microscope. PDGF-B HSPNPs were spread throughout the infarct area, as confirmed with MAP-2 staining at 3 d following injection (Fig. 2*C*).

### PDGF-B HSPNPs reduced the infarct volume and led to better functional recovery

**Figure 3.**
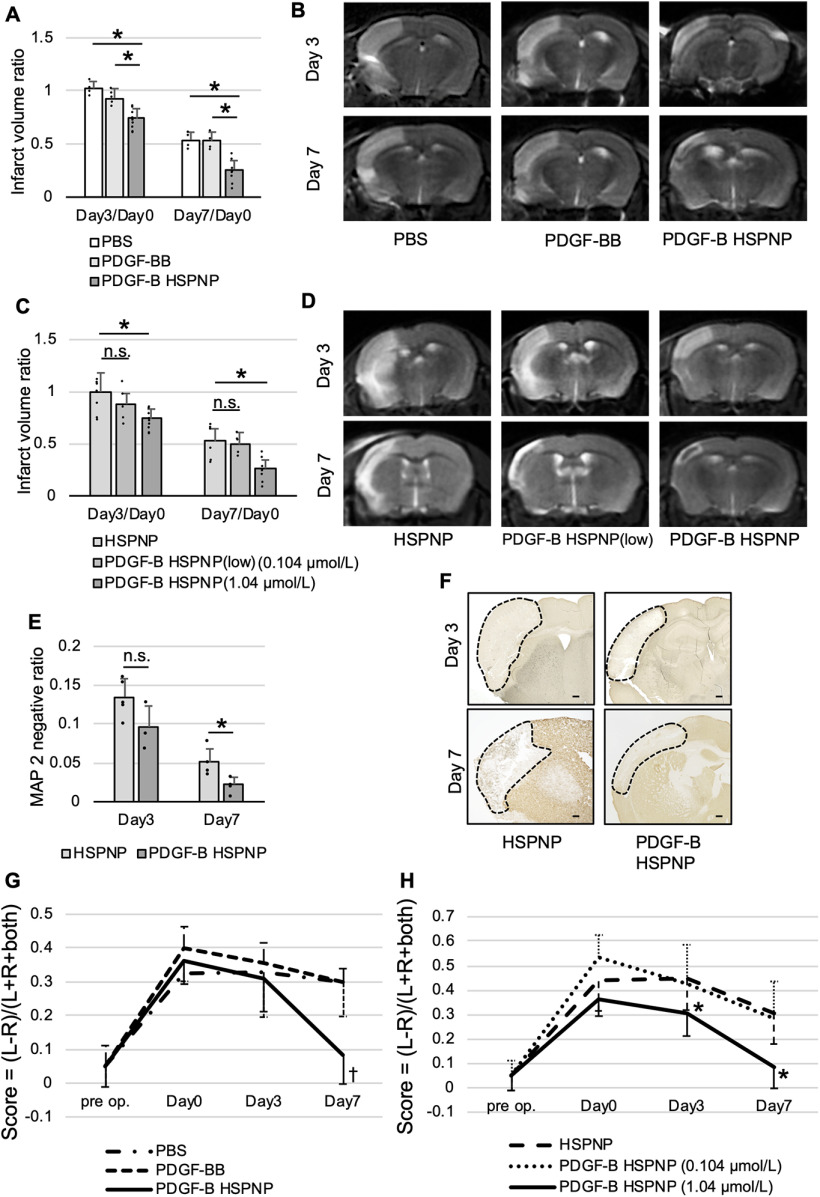
Therapeutic effects of administration of PDGF-B HSPNPs. Evaluation of infarct volume with MRI. The infarct volume was calculated using the following formula: infarct volume ratio = (ipsilateral T2 high intensity area)/(contralateral hemisphere × 2). Because of individual differences in cerebral infarct volume, we evaluated the infarct volume before injection of NPs (24 h after MCAO) as a control. ***A***, The infarct volume decreased in the PDGF-B HSPNP group (1.04 μmol/l) compared with the PBS or PDGF-BB protein group. ***B***, MRI on days 3 and 7 after administration of PBS, PDGF-BB, and PDGF-B HSPNPs. ***C***, The infarct volume decreased in the PDGF-B HSPNP group (1.04 μmol/l) compared with that in the HSPNP group but not in the group administered a lower concentration of PDGF-B HSPNPs (0.104 μmol/l). Shown is mean ± SD (*n* = 5–8; *p < 0.01; n.s., not significant). ***D***, MRI on days 3 and 7 after administration of HSPNPs, PDGF-B HSPNPs (low), and PDGF-B HSPNPs. ***E***, MAP-2 staining demonstrated that the infarct volume decreased in the PDGF-B HSPNP group compared with that in the HSPNP group on day 7. MAP-2-negative ratio = (ipsilateral MAP-2-negative area)/(contralateral hemispheric area × 2). Values are mean ± SD (*n* = 5; *p < 0.05; n.s., not significant). ***F***, MAP-2 staining on days 3 and 7 after administration of HSPNPs and PDGF-B HSPNPs. Scale bars: 100 μm. Dotted areas indicate infarct areas. ***G***, ***H***, Motor functional evaluation using the cylinder test before MCAO, before administration of NPs (day 0), 3 d after administration (day 3), and 7 d after administration (day 7). ***D***, Motor function improved significantly on day 7 in the PDGF-B HSPNP group (1.04 μmol/l) compared with the PBS or PDGF-BB group. ***E***, Motor function improved significantly on day 7 in the PDGF-B HSPNP group (1.04 μmol/l) compared with that in the groups administered HSPNP or lower concentration of PDGF-B HSPNP (0.104 μmol/l). Values are mean ± SD (*n* = 5–8; †*p* < 0.01, PBS and PDGF-BB vs PDGF-B HSPNP), *p < 0.05, HSPNP versus PDGF-B HSPNP.

To investigate the therapeutic effect of PDGF-B HSPNPs, we analyzed the infarct volume and motor function. MRI findings revealed that intravenous administration of PDGF-B HSPNPs (1.04 μmol/l) significantly reduced the infarct volume compared with intravenous administration of PBS or PDGF-BB protein alone on days 3 and 7 (Fig. 3*A*,*B*). The infarct volume did not significantly decrease with administration of lower concentration of PDGF-B HSPNPs (0.104 μmol/l) compared with 1.04 μmol/l PDGF-B HSPNP administration (Fig. 3*C*,*D*). Immunohistochemistry showed that the infarct volume evaluated with MAP-2 staining significantly decreased in the PDGF-B HSPNP group compared with the HSPNP group on day 7 (Fig. 3*E*,*F*).

Next, we investigated the therapeutic effect of PDGF-B HSPNPs on functional recovery using the cylinder test. Motor function significantly improved in the PDGF-B HSPNP group on day 7 compared with the groups that were administered PBS or PDGF-BB (Fig. 3*G*). Additionally, PDGF-B HSPNPs (1.04 μmol/l) significantly improved motor function compared with HSPNPs or lower concentration of PDGF-B HSPNPs (0.104 μmol/l) on days 3 and 7 (Fig. 3*H*). These findings suggested that treatment with PDGF-B HSPNPs (1.04 μmol/l) contributed to decrease in infarct volume and improved functional motor recovery.

### PDGF-B HSPNPs induce phosphorylation of Akt in the ischemic core and peri-infarct areas

**Figure 4.**
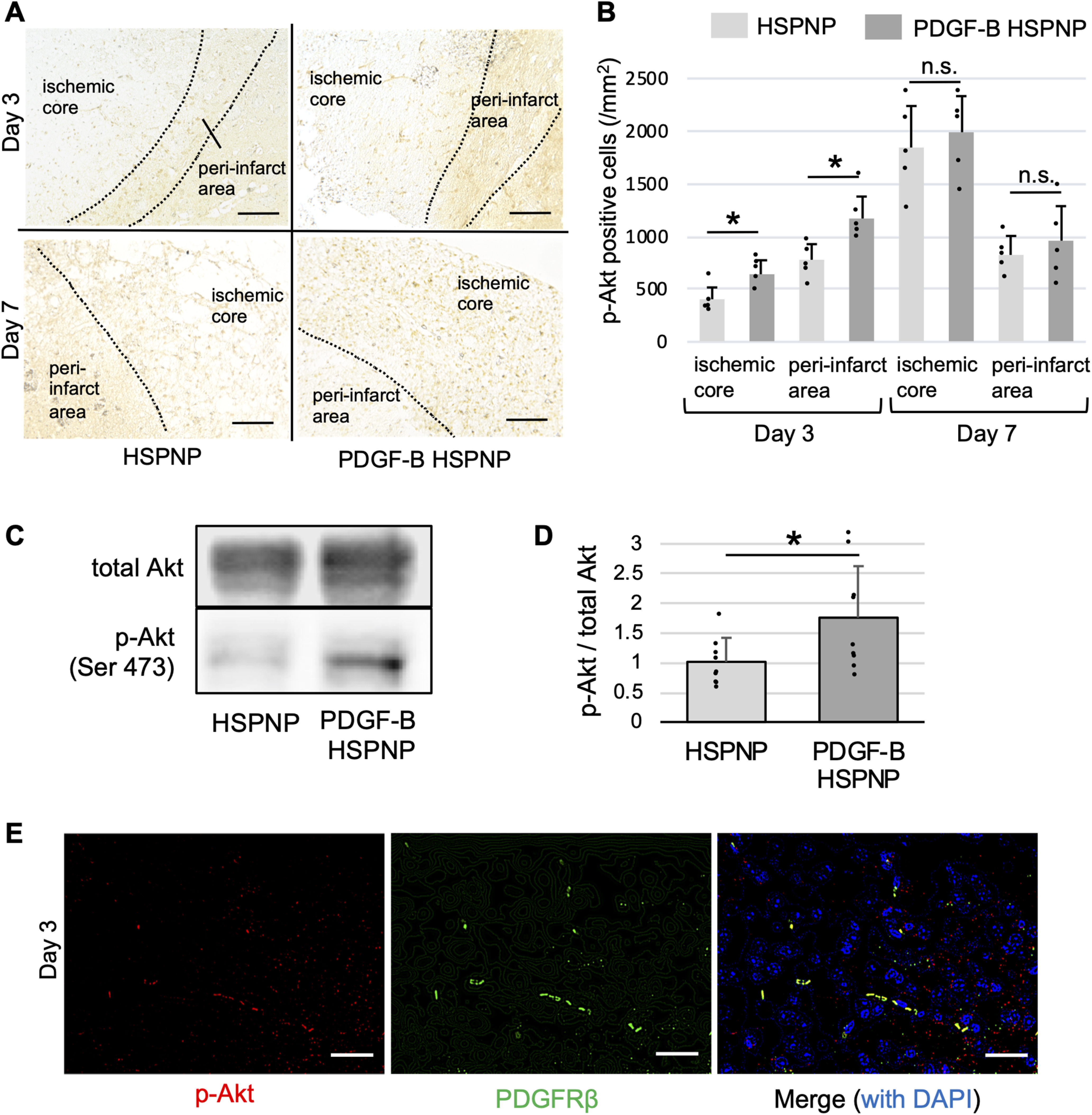
Akt phosphorylation by PDGF-B HSPNPs. ***A***, Temporal profile of phospho-Akt (Ser 473) expression using immunohistochemistry on days 3 and 7 after administration of HSPNPs and PDGF-B HSPNPs. Scale bars: 100 μm. ***B***, Phospho-Akt-positive cells were counted by hybrid cell count in the peri-infarct area and ischemic core. Data are presented as mean ± SD (*n* = 5; *p < 0.05; n.s., not significant). ***C***, Immunoblot analyses for Akt and phospho-Akt (Ser 473) 3 d after administration of HSPNPs and PDGF-B HSPNPs. ***D***, Densitometry for phospho-Akt/total Akt. Data are presented as mean ±SD (*n* = 9; *p < 0.05). ***E***, Immunofluorescent double labeling of phospho-Akt (p-Akt; red) and PDGFRβ (green) in the ischemic core on day 3 after PDGF-B HSPNP administration. Scale bar: 50 μm.

Following up the data obtained from cultured pericytes, we examined whether PDGF-B HSPNPs phosphorylate Akt in vivo through PDGF-B-PDGFRβ signaling. Immunohistochemistry showed significant Akt phosphorylation in the ischemic core and peri-infarct areas on day 3 (Fig. 4*A*,*B*), but there was no significant difference in Akt phosphorylation on day 7 (Fig. 4*B*). These findings suggest that PDGF-B HSPNPs contributed to phosphorylation of Akt on day 3 in the ischemic core and peri-infarct area. Immunoblot analysis consistently demonstrated that Akt was significantly phosphorylated in the PDGF-B HSPNP group compared with the control group (Fig. 4*C*,*D*). Additionally, immunofluorescent double labeling demonstrated that Akt was phosphorylated in PDGFRβ-positive cells on day 3 (Fig. 4*E*). These results suggested that Akt was particularly phosphorylated in PDGFRβ-pericytes 3 d after PDGF-B HSPNP injection.

### PDGF-B HSPNPs increased the production of NTs

**Figure 5.**
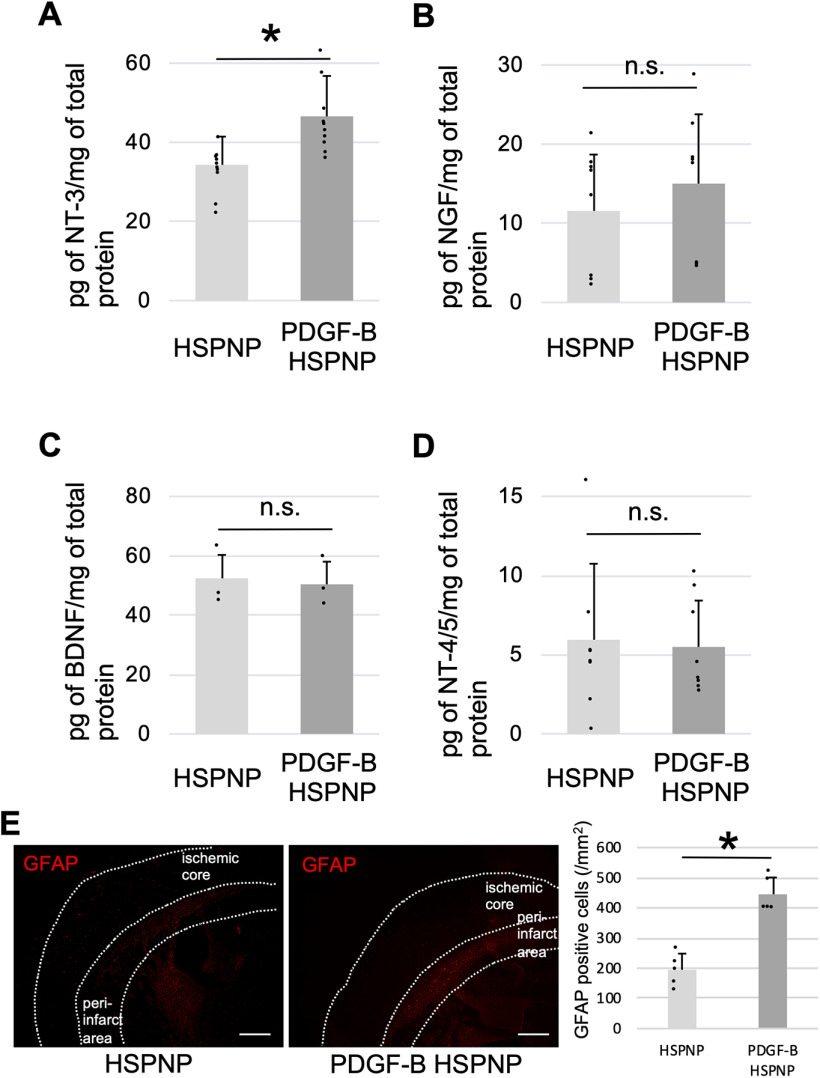
Effects of PDGF-B HSPNPs on the expression of NTs and astrogliosis in peri-infarct area in a murine transient middle cerebral artery occlusion model of stroke. NT expression 3 d after administration of PDGF-B HSPNPs was investigated with ELISA using a multi-NT rapid screening ELISA kit. ***A***, Neurotrophin-3 (NT-3) (*n* = 10; *p < 0.05). ***B***, Nerve growth factor (NGF) (*n* = 8; n.s., not significant.). ***C***, Brain-derived neurotrophic factor (BDNF) (*n* = 8). ***D***, Neurotrophin-4/5 (NT-4/5) (*n* = 8). ***E***, Immunoblot analyses for GFAP 7 d after administration of HSPNPs and PDGF-B HSPNPs. GFAP–positive cells were counted by hybrid cell count in the peri-infarct area. Data are presented as mean ± SD (*p < 0.05).

It was previously reported that PDGF-B-PDGFRβ signaling upregulated the production of NT-3 and NGF in pericytes (Arimura et al., 2012). We examined these factors to investigate the underlying mechanisms of the neuroprotective role of PDGF-B HSPNPs in ischemic stroke. Expression of NT-3 was significantly increased in the PDGF-B HSPNP group compared with the HSPNP group (Fig. 5*A*). NGF was also increased in the PDGF-B HSPNP group, but without a statistically significant difference (Fig. 5*B*). BDNF (Fig. 5*C*), and NT-4/5 (Fig. 5*D*) were similar in the PDGF-B HSPNP and control groups. Our results showed that PDGF-B HSPNPs contribute to increase in NT-3 production after ischemic stroke.

### PDFG-B HSPNPs enhanced astrogliosis in peri-infarct area

It has been reported that pericytes mediate astrogliosis in peri-infarct area after ischemic stroke through PDGF-B signaling (Shibahara et al., 2020). To assess the astrogliosis after ischemia, we examined immunofluorescent staining of GFAP (Fig. 5*E*). Interestingly, expression of GFAP in peri-infarct area on day 7 after treatment was significantly increased in the PDGF-B HSPNP group compared with the HSPNP group. These findings suggested that PDGF-B HSPNPs enhanced astrogliosis in peri-infarct area, which might contribute to the reduction of infarct volume in the PDGF-B HSPNP group compared with the HSPNP group.

### PDGF-B HSPNPs reduced apoptosis in peri-infarct area

**Figure 6.**
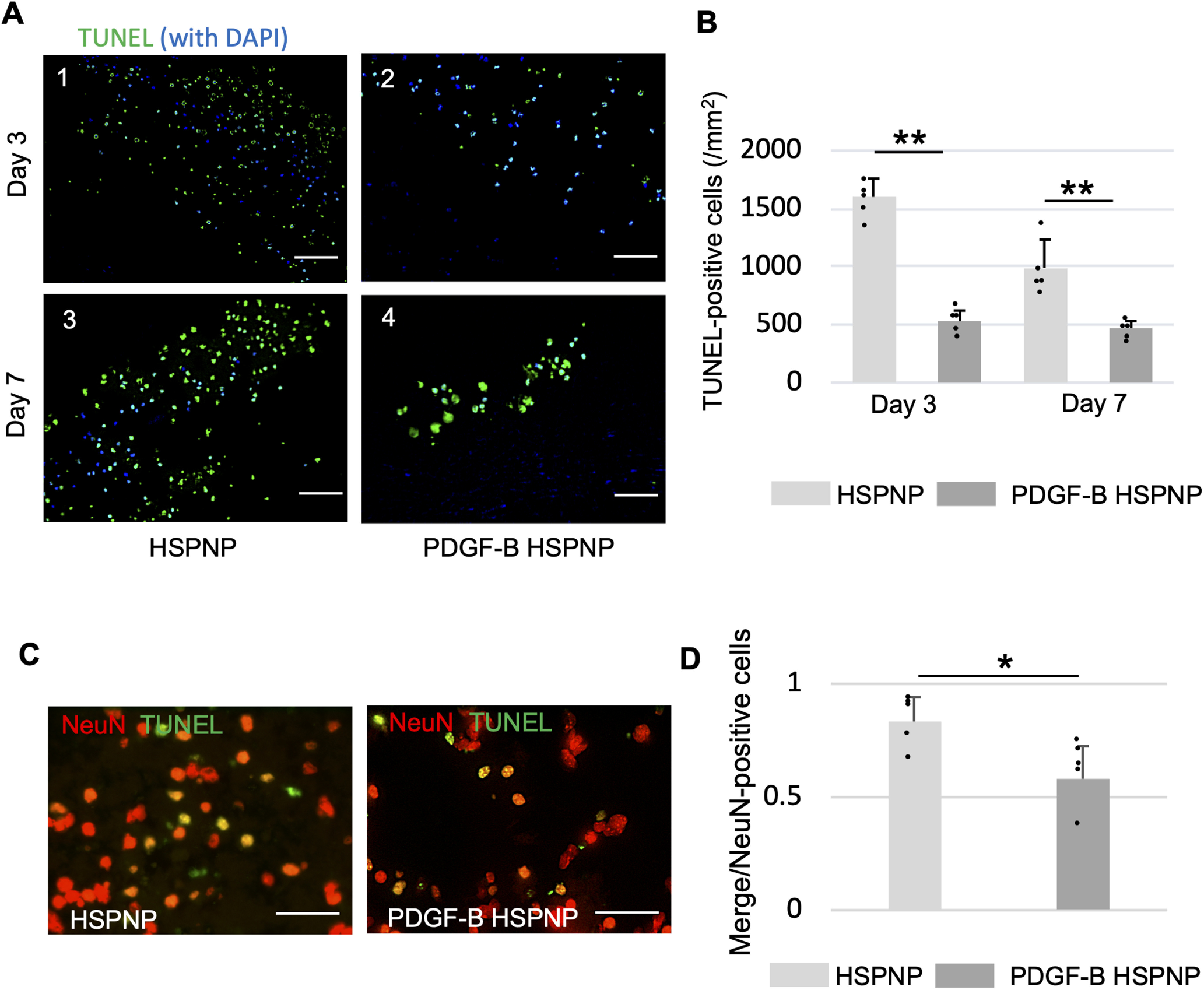
PDGF-B HSPNPs reduced neuronal apoptosis in peri-infarct area. ***A***, TUNEL staining with DAPI in peri-infarct area; 1 and 3 are representative images of the HSPNP group, and 2 and 4 are images of the PDGF-B HSPNP group (1 and 2: 3 d after administration; 3 and 4: 7 d after administration). Scale bar: 100 μm. ***B***, The number of TUNEL-positive cells in peri-infarct area 3 and 7 d after administration of HSPNPs and PDGF-B HSPNPs (*n* = 5 for each group; **p < 0.01). ***C***, Fluorescent TUNEL/NeuN (green/red, respectively) double staining 3 d after administration of HSPNPs (left) and PDGF-B HSPNPs (right). Scale bar: 50 μm. ***D***, The number of TUNEL/NeuN double-positive cells scaled to the number of NeuN-positive cells in peri-infarct area 3 d after administration of HSPNPs and PDGF-B HSPNPs (*n* = 5 for each group; *p < 0.05).

TUNEL staining was performed to assess apoptosis in peri-infarct area after ischemic stroke. Three and 7 d after administration, the total number of apoptotic cells in peri-infarct area was significantly lower in the PDGF-B HSPNP group compared with those in the HSPNP group (Fig. 6*A*,*B*). Furthermore, TUNEL staining and NeuN (red) staining were used for fluorescent double staining to identify cells that escaped apoptosis. TUNEL-NeuN double-positive cells represented apoptotic neurons. In peri-infarct area, the proportion of apoptotic neuronal cells was significantly lower in the PDGF-B HSPNP group (Fig. 6*C*,*D*). These results indicated that PDGF-B HSPNP administration suppressed neuronal cell apoptosis in peri-infarct area.

## Discussion

This study showed that PDGF-B HSPNPs contribute to decreased infarct volume and better functional recovery by reducing neuronal apoptosis in a t-MCAO mouse model (t-MCAO). PDGF-B HSPNPs administered 24 h after t-MCAO accumulated in the infarct area 3–7 d after injection via the tail vein. In the PDGF-B HSPNP group, Akt was significantly phosphorylated within pericytes in the infarct area. NT-3 production and an anti-apoptotic effect were enhanced by PDGF-B HSPNPs, which may contribute to reduced infarct volume. Moreover, astrogliosis in peri-infarct area was enhanced by PDGF-B HSPNPs. These results suggested that treatment targeted at enhancing PDGF-B signaling in pericytes in an infarct area, using a DDS, may be effective for ischemic stroke.

DDSs using nanomaterials have been developed for the treatment of cancer, infection, and vascular diseases (Wilczewska et al., 2012); however, there have been few reports of DDSs for central nervous system diseases. This is mainly because of the poor permeability of drugs or materials through the BBB. The BBB comprises endothelial cells, pericytes, astrocytes, microglia, and the basal lamina and contributes to the maintenance of brain homeostasis through the regulation of vascular permeability. As a result, drugs are restricted from moving into the brain. A previous report showed reduced infarct volume through the administration of fullerene NPs in a rodent cerebral infarction model (Vani et al., 2016). In addition, it has been reported that administration of radical scavenger-containing NPs (Hosoo et al., 2017) or rutin-encapsulated chitosan NPs (Ahmad et al., 2016) immediately after reperfusion results in decreased infarct volume and improved neurologic findings. In our study, we were able to confirm that our NPs (HSPNPs) accumulated in the infarct area from 6 h to one week after administration. Although the mechanism of HSPNP accumulation within ischemic lesions has not been completely elucidated, the enhanced permeability and retention effect is considered a potential mechanism because the permeability of the BBB is increased after infarction. In contrast, another report found that NPs administered after 6 h of cerebral ischemia did not reach the infarct area (Ishii et al., 2012). This might be attributable to the large particle size used in that study (129 nm AEPO-liposomes using PEGylated liposomes). The HSPNPs used in our study were much smaller, which may explain the distribution of our HSPNPs within the infarct area. Moreover, we confirmed the retention of HSPNPs in the infarct area at least one week after injection. In studies using HSPNPs as DDSs for tumors (Kim et al., 1998; Murata et al., 2015; Kawano et al., 2018), the distribution of HSPNPs after intravenous injection showed accumulation in the liver and rapid excretion from the kidney. The half-life of HSPNPs was 12–18 h, and no toxicity was observed by biological evaluation. The retention of PDGF-B HSPNPs may contribute to the sustainable upregulation of PDGF-B-Akt signaling in pericytes and neuroprotection through the upregulation of NT.

Pericytes play an important role after ischemic stroke in relation to angiogenesis, maintenance of the BBB, regulation of blood flow, and tissue repair, as well as exhibiting neurotrophic effects. A previous study reported that pericytes were upregulated on days 3–7 in areas of infarction and had neurotrophic effects through PDGF-B-Akt signaling after ischemic stroke. We have reported previously that PDGF-B-Akt signaling in brain pericytes promoted the expression of NTs such as NT-3 and NGF (Arimura et al., 2012). Additionally, it has been reported that extrinsic application of NT-3 may be associated with protection of nerve cells from ischemic damage and inhibition of DNA fragmentation (Zhang et al., 1999). In the present study, we confirmed that PDGF-B HSPNP administration significantly increased the phosphorylation of Akt and the expression of NT-3. Therefore, it may be speculated that the neuroprotective effect of PDGF-B HSPNP may be partially mediated by the production of NTs through PDGF-B-Akt signaling after ischemic stroke. We also compared intravenous administration of PDGF-BB with PDGF-B HSPNP administration, but intravenous administration of PDGF-BB alone did not reduce the infarct volume or improve neurologic function. A previous report suggested that neither PDGF-AA nor PDGF-BB reached brain tissue via intravenous injection because of the instability and protein binding in circulating blood (Kastin et al., 2003). Therefore, our method using HSPNPs is useful to deliver the PDGF protein to the ischemic lesion and efficiently activate PDGF-B signaling.

We also demonstrated that an anti-apoptotic effect was activated with the injection of PDGF-B HSPNPs 1 d after t-MCAO. Although necrotic cell death begins in the ischemic core because of the dramatic flow reduction in ischemic stroke, there is a recoverable region known as the ischemic penumbra with preserved metabolism surrounding the ischemic core. Recent studies have reported that many neurons in the ischemic penumbra might undergo apoptosis after several hours or days. This programmed cell death should be the therapeutic target in ischemic stroke, but effective treatment is lacking other than reperfusion therapy, which has a narrow therapeutic time window, using thrombolytic agent or mechanical clot retrievers. Apoptotic cell death sustained for 48 h after t-MCAO was shown in rodent models (Zhang et al., 2010; Yamashita and Abe, 2016), suggesting that there is a therapeutic time window to reduce apoptotic neuronal death until 48 h after ischemic injury. In the present study, it was revealed that apoptotic neuronal death decreased in the PDGF-B HSPNP group compared with the non-modified HSPNP group. These results may lead to a novel neuroprotective approach using DDS with wide therapeutic time window for ischemic stroke. However, the mechanism of the anti-apoptotic effect with PDGF-B HSPNPs was not fully elucidated. We confirmed the activation of Akt signaling in pericytes in the ischemic region and increased production of neurotrophic factors, but there may be another mechanism implicated in the PDGF-B HSPNP anti-apoptotic effect. Since Akt is well known as a key regulator of neuronal cell apoptosis, there is a possibility that PDGF-B HSPNPs reacted to cells other than pericytes (Kilic et al., 2005).

In addition, PDGF-B signaling in pericytes is important for intra-infarct fibrosis and peri-infarct astrogliosis after ischemic stroke (Tachibana et al., 2017; Shibahara et al., 2020). In the present study, expression of GFAP in the peri-infarct area was significantly upregulated after administration of PDGF-B HSPNPs (Fig. 5*E*). These results suggested that astrogliosis in the peri-infarct area was upregulated by PDGF-B HSPNPs through PDGF-B signaling in pericytes. The reduction of ischemic volume and functional recovery were more evident at day 7 after PDGF-B HSPNP treatment compared with day 3. We speculate that promoting astrogliosis in the peri-infarct area by PDGF-B HSPNPs may contribute to the reduction of ischemic volume and functional recovery after acute phase. Additionally, PDGF-B signaling in pericytes is important for maintaining the BBB, recovering blood flow inside the infarction, and regulating blood flow. As these neuroprotective effects of PDGF-B signaling in pericytes could possibly contribute to the recovery after ischemic stroke, further investigation is needed to elucidate the detailed mechanisms.

This study has several limitations. First, because the study involved only one week of follow-up, the long-term effects and toxicity of PDGF-B HSPNPs remain unknown. Second, we did not verify the pharmacodynamics of PDGF-B HSPNPs in vivo. We aim to perform further studies to develop a clinical use for this drug. Third, the detailed mechanisms of the neuroprotective effect of PDGF-B HSPNPs have not been fully elucidated as mentioned above. Although we demonstrated the anti-apoptotic effect and promoting astrogliosis by PDGF-B HSPNPs, further investigation from a different perspective is needed. Fourth, as we used the t-MCAO mouse model, the effect of PDGF-B HSPNPs in permanent vessel occlusion is unknown. To apply this novel therapy using DDS for all cases of ischemic stroke in humans, we should investigate the pharmacodynamics in permanent ischemic models. Finally, as we used a distal MCAO model, the ischemic volume was relatively small and the basal ganglia were not part of the infarct area. We used a single distal t-MCAO rodent model that cannot replicate the pathologic conditions of large-vessel occlusion in humans. We should investigate the efficacy of PDGF-B HSPNPs for large infarctions in the future.

In conclusion, PDGF-B HSPNPs reduced the infarct volume and improved motor functional recovery through an anti-apoptotic effect and promoting astrogliosis in peri-infarct area after ischemic stroke. These results suggest that treatment with PDGF-B HSPNPs may be a novel approach for ischemic stroke, although further studies are needed to validate these findings.

## Synthesis

Reviewing Editor: Christie Fowler, University of California, Irvine Decisions are customarily a result of the Reviewing Editor and the peer reviewers coming together and discussing their recommendations until a consensus is reached. When revisions are invited, a fact-based synthesis statement explaining their decision and outlining what is needed to prepare a revision will be listed below. The following reviewer(s) agreed to reveal their identity: Sowmya Yelamanchili.

The manuscript has returned from peer review by two experts in the field. After consultation, both reviewers identified value for these studies related to their potential impact on the field. However, several points were identified that require revision prior to acceptance of the manuscript. These include:

1. The authors should be more restrictive in using the term “tissue repair”. The authors seem to use confusingly the term “tissue repair” to astrogliosis: for example, “tissue repair through astrogliosis in peri-infarct area” in the abstract and other places. PDGFRβ-mediated fibrotic tissue repair occurs within infarct area, while astrogliosis does in peri-infarct areas. The authors should clearly differentiate astrogliosis from potential intra-infarct tissue repair. The papers (Tachibana, et al. and Shibahara, et al.) cited by the authors demonstrate that PDGFRβ-mediated fibrotic repair within infarct areas promotes peri-infarct astrogliosis, a marker of neural reorganization, through production of trophic factors and leads to functional recovery. These papers do not demonstrate that peri-infarct astrogliosis promote intra-infarct tissue repair.

2. The following phrase should be deleted: “possibly because of decrease in the infarct volume” in Page 26, line 466. The reduction of apoptotic cell death in peri-infarct areas is not directly relevant to temporal reduction of infarct volume.

3. The visual abstract and significance statement need to be revised in consideration of the above two concerns.

## Author Response

We appreciate your and the reviewers' insightful feedback on our paper. We have incorporated changes that reflect the detailed suggestions you have provided. We hope that our edits and the responses provided below satisfactorily address all the issues and concerns you and the reviewers have noted.

The following is a point-by-point response to the questions and comments delivered in your letter dated July 18, 2021.

Synthesis of Reviews:

Computational Neuroscience Model Code Accessibility Comments for Author (Required):

N/A

Significance Statement Comments for Author (Required):

Need to rephrase statement regarding tissue repair and astrogliosis - please see below

Comments on the Visual Abstract for Author (Required):

Need to modify - please see below

Synthesis Statement for Author (Required):

The manuscript has returned from peer review by two experts in the field. After consultation, both reviewers identified value for these studies related to their potential impact on the field. However, several points were identified that require revision prior to acceptance of the manuscript. These include:

1. The authors should be more restrictive in using the term “tissue repair”. The authors seem to use confusingly the term “tissue repair” to astrogliosis: for example, “tissue repair through astrogliosis in peri-infarct area” in the abstract and other places. PDGFRβ-mediated fibrotic tissue repair occurs within infarct area, while astrogliosis does in peri-infarct areas. The authors should clearly differentiate astrogliosis from potential intra-infarct tissue repair. The papers (Tachibana, et al. and Shibahara, et al.) cited by the authors demonstrate that PDGFRβ-mediated fibrotic repair within infarct areas promotes peri-infarct astrogliosis, a marker of neural reorganization, through production of trophic factors and leads to functional recovery. These papers do not demonstrate that peri-infarct astrogliosis promote intra-infarct tissue repair.

2. The following phrase should be deleted: “possibly because of decrease in the infarct volume” in Page 26, line 466. The reduction of apoptotic cell death in peri-infarct areas is not directly relevant to temporal reduction of infarct volume.

3. The visual abstract and significance statement need to be revised in consideration of the above two concerns.

Response: Thank you for the valuable comment. As you pointed out, we should clearly differentiate astrgliosis. Following your suggestion, we have added an additional description in significance statement and visual abstract, and deleted: “possibly because of decrease in the infarct volume” in Page 26, line 466.

(Revised) Visual abstract

We have modified “astrogliosis in peri-infarct area", and deleted “fibrotic tissue repair”.

Abstract

We have deleted: “PDGF-B HSPNPs activate PDGF-B-Akt signaling in infarct areas, which plays an important neuroprotective role after cerebral infarction.” in Page 1, line 17-19.

"Moreover, astrogliosis in peri-infarct area was significantly upregulated with PDGF-B HSPNPs compared with HSPNPs alone.” (page 1, lines 17-18)

Significance Statement

"Additionally, it has been revealed that astrogliosis in peri-infarct area was also promoted with PDGF-B HSPNPs.” (page 2, lines 28-29)

“It has been reported that pericytes mediate astrogliosis in peri-infarct area after ischemic stroke through PDGF-B signaling (Shibahara et al., 2020).” (page 21, lines 378-379)

“These findings suggested that PDGF-B HSPNPs enhanced astrogliosis in peri-infarct area, which might contribute to the reduction of infarct volume in the PDGF-B HSPNP group compared with the HSPNP group.” (page 21, lines 383-385)

“In the present study, it was revealed that apoptotic neuronal death decreased in the PDGF-B HSPNP group compared with the non-modified HSPNP group.” (page 26, lines 465-467)

"In addition, PDGF-B signaling in pericytes is important for intra-infarct fibrosis and peri-infarct astrogliosis after ischemic stroke (Tachibana et al., 2017; Shibahara et al., 2020).” (page 27, lines 475-477)

"We speculate that promoting astrogliosis in the peri-infarct area by PDGF-B HSPNPs may contribute to the reduction of ischemic volume and functional recovery after acute phase.” (page 27, lines 481-483)

"Although we demonstrated the anti-apoptotic effect and promoting astrogliosis by PDGF-B HSPNPs, further investigation from a different perspective is needed.” (page 28, lines 493-495)

"PDGF-B HSPNPs reduced the infarct volume and improved motor functional recovery through an anti-apoptotic effect and promoting astrogliosis in peri-infarct area after ischemic stroke.” (page 28, lines 505-507)
