# Erratum: Takagishi et al., “Protein Nanoparticles Modified with PDGF-B as a Novel Therapy after Acute Cerebral Infarction”

**DOI:** 10.1523/ENEURO.0143-24.2024

**Published:** 2024-04-08

In the article “Protein Nanoparticles Modified with PDGF-B as a Novel Therapy after Acute Cerebral Infarction,” by Soh Takagishi, Koichi Arimura, Masaharu Murata, Katsuma Iwaki, Tomohiro Okuda, Keisuke Ido, Ataru Nishimura, Sayoko Narahara, Takahito Kawano, and Koji Iihara which published online on August 30, 2021, Figure 3 and its legend appeared incorrectly. In Figure 3*B*, an incorrect image was used for the lower-middle panel. Additionally, the legend for *G* and *H* were labeled incorrectly as *D* and *E*. The correct figure and legend appear below. This does not affect the conclusions of the paper.

**Figure 3.**
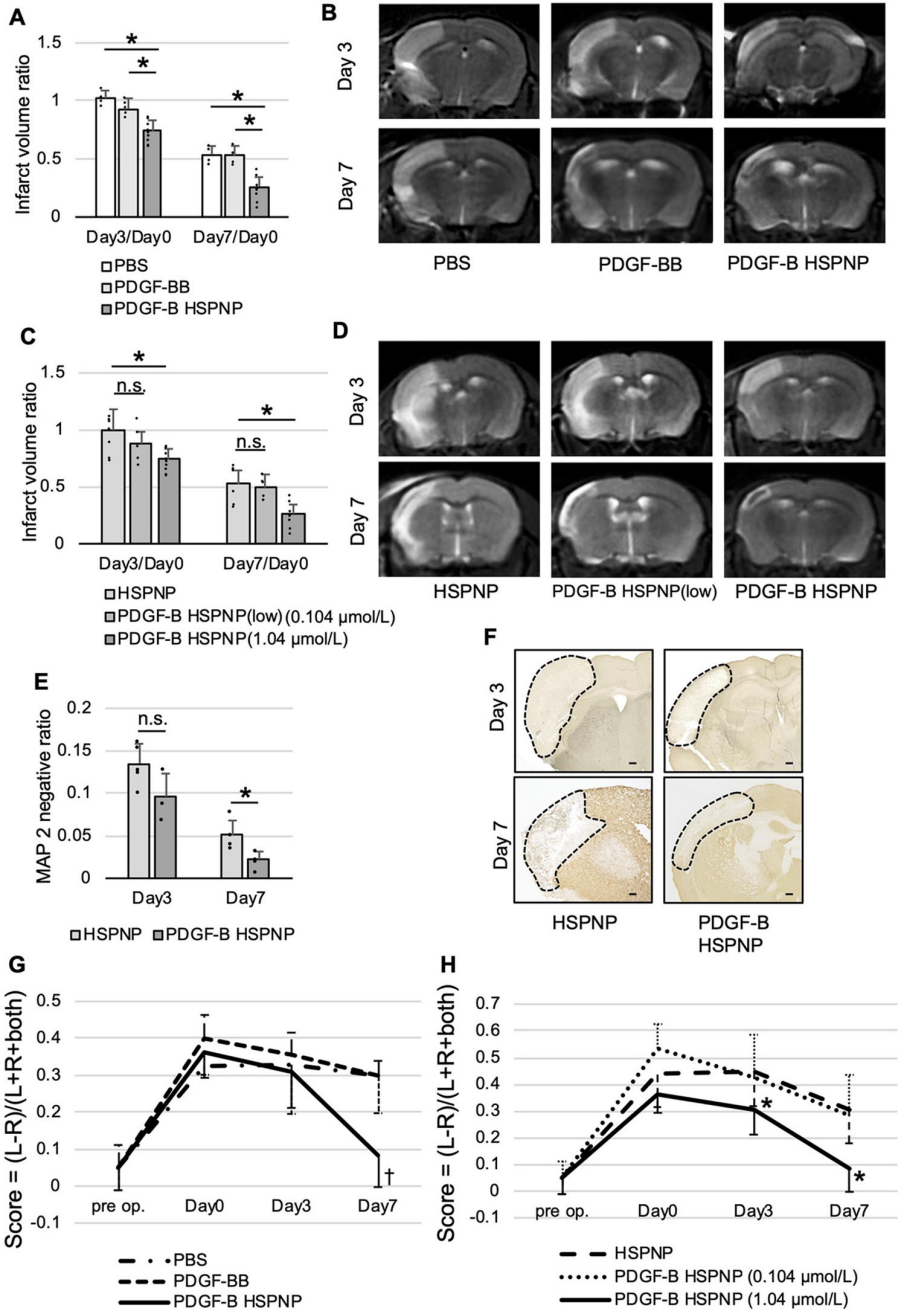
Therapeutic effects of administration of PDGF-B HSPNPs. Evaluation of infarct volume with MRI. The infarct volume was calculated using the following formula: infarct volume ratio = (ipsilateral T2 high intensity area) / (contralateral hemisphere × 2). Because of individual differences in cerebral infarct volume, we evaluated the infarct volume before injection of NPs (24 h after MCAO) as a control. ***A***, The infarct volume decreased in the PDGF-B HSPNP group (1.04 µmol/L) compared with the PBS or PDGF-BB protein group. ***B***, MRI on Days 3 and 7 after administration of PBS, PDGF-BB, and PDGF-B HSPNPs. ***C***, The infarct volume decreased in the PDGF-B HSPNP group (1.04 µmol/L) compared with that in the HSPNP group but not in the group administered a lower concentration of PDGF-B HSPNPs (0.104 µmol/L). Shown is mean ± SD (*n* = 5–8; *p < 0.01; n.s., not significant). ***D***, MRI on Days 3 and 7 after administration of HSPNPs, PDGF-B HSPNPs (low), and PDGF-B HSPNPs. ***E***, MAP-2 staining demonstrated that the infarct volume decreased in the PDGF-B HSPNP group compared with that in the HSPNP group on Day 7. MAP-2-negative ratio = (ipsilateral MAP-2-negative area) / (contralateral hemispheric area × 2). Values are mean ± SD (*n* = 5; *p < 0.05; n.s., not significant). ***F***, MAP-2 staining on Days 3 and 7 after administration of HSPNPs and PDGF-B HSPNPs. Scale bar: 100 µm. Dotted areas indicate infarct areas. ***G***, ***H***, Motor functional evaluation using the cylinder test before MCAO, before administration of NPs (Day 0), 3 d after administration (Day 3), and 7 d after administration (Day 7). ***G***, Motor function improved significantly on Day 7 in the PDGF-B HSPNP group (1.04 µmol/L) compared with the PBS or PDGF-BB group. ***H***, Motor function improved significantly on Day 7 in the PDGF-B HSPNP group (1.04 µmol/L) compared with that in the groups administered HSPNP or lower concentration of PDGF-B HSPNP (0.104 µmol/L). Values are mean ± SD (*n* = 5–8; ^†^*p* < 0.01, PBS and PDGF-BB vs PDGF-B HSPNP), *p < 0.05, HSPNP vs PDGF-B HSPNP.

